# Serum VEGF, high-sensitivity CRP, and cystatin-C assist in the diagnosis of type 2 diabetic retinopathy complicated with hyperuricemia

**DOI:** 10.1515/med-2023-0857

**Published:** 2023-12-07

**Authors:** Jing Wei, Jincheng Zhang, Yanan Shi, Huiqin Zhang, Yan Wu

**Affiliations:** No.1 Department of Endocrinology, Cangzhou Central Hospital, Cangzhou City, Hebei Province, 061000, China; No.1 Department of Ophthalmology, Cangzhou Central Hospital, Cangzhou City, Hebei Province, 061000, China; No.1 Department of Endocrinology, Cangzhou Central Hospital, 16 West Xinhua Road, Cangzhou City, Hebei Province, 061000, China

**Keywords:** type 2 diabetes mellitus, retinopathy, hyperuricemia, vascular endothelial growth factor, inflammation, high-sensitivity C-reactive protein, cystatin C, Pearson test, receiver operating characteristic curve, logistic multiple factor regression analysis

## Abstract

Elevated serum uric acid (UA) level is related to type 2 diabetic retinopathy (DR). Vascular endothelial growth factor (VEGF), high-sensitivity C-reactive protein (hs-CRP), and cystatin C (Cys-C) have involvement in type 2 DR complicated with hyperuricemia (HUA) (HUDR), and we explored their clinical values in HUDR. Type 2 DR patients were allocated into HUDR/DR groups, with type 2 diabetes mellitus (T2DM) patients as the control group. Serum VEGF and inflammation markers hs-CRP, and Cys-C levels were assessed by ELISA and immunoturbidimetry. The correlations between serum UA level and VEGF/hs-CRP/Cys-C were analyzed by Pearson tests, diagnostic values of VEGF/hs-CRP/Cys-C were analyzed by receiver operating characteristic curves, and the independent risk factors in HUDR were analyzed by logistic multivariate regression. Serum VEGF/hs-CRP/Cys-C level differences among the T2DM/DR/HUDR groups were statistically significant, with the levels in HUDR > DR > T2DM. Serum UA level in HUDR patients was positively correlated with serum VEGF/hs-CRP/Cys-C. Serum VEGF/hs-CRP/Cys-C assisted in HUDR diagnosis, with their combination showing the greatest diagnostic value. UA/FPG/HbA1C/VEGF/hs-CRP/Cys-C were independent risk factors for HUDR. The incidence of proliferative DR was increased in HUDR patients. Collectively, serum VEGF, hs-CRP, and Cys-C levels in HUDR patients were increased, and HUA might promote DR progression.

## 1 Introduction

Diabetic retinopathy (DR) is the most prevalent complication of type 2 diabetes mellitus (T2DM) identified as a microvascular disease [1]. Its occurrence is closely linked to the disease course and blood glucose level [2], both of which are characterized by an aggravated burden of inflammation [3,4]. DR is a leading cause of blindness among diabetic individuals [5], whose incidence rate is increasing worldwide and has become a global public health issue [6]. Besides, it has been revealed that people with DR is at raised risk for systemic vascular complications, comprising coronary heart disease, subclinical and clinical stroke, nephropathy, and heart failure [7]. Hyperuricemia (HUA) is a condition in which the serum uric acid (UA) levels are elevated caused by purine metabolic abnormalities, mainly due to the increased formation or reduced excretion of UA [8]. Serum UA and UA-based markers are bound up with inflammatory diseases such as T2DM [9], thyroiditis [10], diabetic kidney injury (DKI) [11], fatty degeneration of liver [12], hypertension [13], and metabolic syndrome [14]. Males with serum UA ≥420 μmol/L or females with UA ≥360 μmol/L can be diagnosed with HUA [15]. HUA has the ability to activate oxidative stress reaction and encourage the release of inflammatory factors [16], whereas a variety of inflammatory factors and oxidative stress play an essential role in diabetic microvascular disease. Research reveals that HUA is an independent risk factor for T2DM and DR [17,18]. With a rapidly aging population worldwide, increasing lifespan, and lifestyle changes, there is an elevated risk for DM and a higher burden of DR and need for eye treatment and care, and accurate and up-to-date estimation of DR prevalence is vital for health policy formulation and disease management [6]. Therefore, it is essential to study the relationship between DR and HUA and find efficient targets for DR alleviation.

Our understanding of vascular endothelial growth factor (VEGF), the main player in DR pathogenesis, has developed over the years, which results in a paradigm shift in DR understanding and its treatment [19]. It is also interrelated to inflammation, such as that serum VEGF is reportedly highly expressed in patients with atopic dermatitis (chronic inflammatory disease) [20]. Furthermore, T2DM patients who received vitamins along with antidiabetic medication manifest a slower rate of DR development and decreased abnormal biochemical mediators such as VEGF, malondialdehyde, and reactive oxygen species [21]. DR begins with the abnormalities in asymptomatic retina and may develop into advanced-stage proliferative DR (PDR), which is characterized by preretinal/vitreous hemorrhages or neovascularization [22]. VEGF-mediated angiogenesis is a vital compensatory response to the microvascular rarefaction in the diabetic retina, which can cause PDR [19]. C-reactive protein (CRP) is an acute-phase protein and an inflammatory cytokine that induces retinal VEGF that is involved in the progression of PDR and mainly synthesized by adipose tissue or liver when tissue injury or microbial invasion occurs [23,24]. Raised blood levels of CRP are related to T2DM and its complications, including DR [25]. High-sensitivity CRP (hs-CRP), a helpful biomarker for determining inflammatory levels [26], owns a certain facilitation on the inflammatory response in the occurrence and progression of DR. Through stimulation of certain organs or tissues, severe inflammatory responses can occur, ultimately causing the occurrence and progression of DR. Cystatin C (Cys-C) is a 13 kDa cysteine proteinase inhibitor protein produced at a steady rate by all nucleated cells and established as an accurate biomarker of chronic kidney disease [27]. Serum Cys-C is considered a novel biomarker for the diagnosis of diabetic nephropathy [28]. It has been previously reported that serum Cys-C level can be viewed as inflammation markers to forecast the death rate and severity of inflammatory diseases [29]. Higher serum Cys-C levels are positively associated with the frequency of DR, chronic heart disease, and stroke in type 2 DM patients with normal renal function or mild renal impairment [30]. However, there are few studies on the relationship between UA and DR, and the clinical significance of serum VEGF, hs-CRP, and Cys-C in type 2 DR patients complicated with HUA (HUDR). This study aims to investigate the possible mechanism of UA in DR by analyzing the levels of serum VEGF, hs-CRP, and Cys-C in type 2 HUDR patients, and their relationships with UA.

## 2 Materials and methods

### 2.1 Study subjects

A total of 200 patients with type 2 DR admitted to Cangzhou Central Hospital from January 2021 to April 2022 were selected as the study subjects. According to the serum UA level, the subjects were assigned to the HUDR group and non-HUDR group (DR group), with 100 in each group. Another 100 T2DM patients (T2DM group) were selected as the control group. T2DM was diagnosed according to the standard of the American Diabetes Association (2019) [31].


**Ethical approval and consent to participate:** The experiments were authorized by the academic ethics committee of Cangzhou Central Hospital (Approval number: 2020-224-01(Z)). All procedures were strictly implemented according to *Declaration of Helsinki*. All subjects involved were fully informed of the study objective and signed the informed consent before sampling.

### 2.2 Inclusion and exclusion criteria

Inclusion criteria were as follows: (1) meeting the criteria of T2DM diagnosis, (2) going through the DR screening and diagnosed with DR for the first time by fundus angiography following the international clinical DR severity grading standard in 2002, (3) a definite diagnosis of HUA, with the male serum UA ≥420 μmol/L and female UA ≥360 μmol/L [15]; (4) 18–75 years old, and (5) with clear and complete clinical data.

Exclusion criteria were as follows: (1) type I DM, gestational diabetes, or other special types of DM; (2) with acute complications of diabetes and could cause HUA; (3) secondary HUA caused by renal excretion disorder, urethral obstruction, or other reasons; (4) the use of diuretics, benzbromarone, and other drug known to affect UA metabolism; (5) with retinopathy caused by other reasons; (6) complicated with serious heart, liver, kidney, lung and other diseases or immune diseases, and malignant tumors; and (7) with stress reactions that caused the inflammatory response of the body, such as trauma and hypoxia.

### 2.3 DR diagnosis

All the subjects accepted standardized clinical ophthalmic examinations conducted by experienced ophthalmologists, including ophthalmic history review, vision and intra ocular pressure measurement, slit lamp examination, and expanded fundus examination [15]. According to the results of ophthalmic examination, DR patients were allocated into non-proliferative DR (NPDR) and PDR groups.

### 2.4 Data and sample collection

The following general clinical data were collected by questionnaire: age, gender (male/female), body mass index (BMI), duration of diabetes (years), systolic blood pressure (SBP), diastolic blood pressure (DBP), smoking and drinking history, hypertension, and coronary heart disease history. After admission to the hospital, 10 mL of fasting peripheral venous blood was collected in the procoagulant tubes [32] in the morning of the next day and centrifuged at 3,500 rpm for 5 min after standing for 2 h in the laboratory of the hospital. Then, the supernatant was collected to determine serum UA, fasting blood glucose (FPG), fasting insulin, fasting C-peptide, creatinine, urea nitrogen (UN), total cholesterol (TC), triglyceride (TG), low-density lipoprotein cholesterol (LDL-C), high-density lipoprotein cholesterol (HDL-C), and hemoglobin A1C (HbA1C) [15,33,34]. FPG was assessed by the glucose oxidase method, HbA1C was assessed by an automatic glycosylated hemoglobin analyzer (Golon Technology, Guangzhou, Guangdong, China) based on high-performance liquid chromatography, blood lipid, and UA, creatinine, UN, and other biochemical indicators were assessed by an automatic biochemical analyzer (Roche Diagnostics, Basel, Switzerland), and fasting insulin and fasting C-peptide were assessed by automatic chemiluminescence immunoassay (BIOBASE). Serum Cys-C level was evaluated by particle-enhanced immunoturbidimetry using a Cobas 8000 c702 analyzer (Roche Diagnostics) [30,32].

### 2.5 Enzyme-linked immunosorbent assay (ELISA)

Serum VEGF (ml064281) and hs-CRP (ml092638) levels of patients were determined using the ELISA kit (mlbio, Shanghai, China) according to the instructions [35].

### 2.6 Statistical analysis

SPSS 21.0 (IBM Corp. Armonk, NY, USA) and GraphPad Prism 8 (GraphPad Software Inc., San Diego, CA, USA) statistical software were employed to analyze and map the data. The Kolmogorov Smirnov test showed that the data were in a normal distribution, and the data were expressed as mean ± standard deviation. Data between the two groups were compared by unpaired *t*-test, data among multi-groups were compared by one-way analysis of variance (ANOVA), followed by Tukey’s multiple comparisons test. The categorical variables were analyzed by Fisher’s exact test. The correlations between serum UA level and VEGF, hs-CRP, and Cys-C were analyzed by Pearson tests. The diagnostic values of serum VEGF, hs-CRP, and Cys-C in DR were analyzed by the receiver operating characteristic curve (ROC). The differences in the area under the ROC curve (AUC) were compared using the MedCalc software. The risk factors affecting DR were evaluated by Logistic multivariate regression analysis. *P* < 0.05 was indicative of statistical significance.

## 3 Results

### 3.1 Baseline characteristics

A total of 300 patients with T2DM were enrolled, including 100 patients in each of the HUDR, DR, and T2DM groups. The comparative analysis of clinical baseline data of the three groups manifested that there were no significant differences in the three groups in terms of age, gender, BMI, SBP, DBP, smoking and drinking history, hypertension, coronary heart disease, fasting insulin, fasting C-peptide, creatinine, TC, TG, LDL-C, and HDL-C (all *P* > 0.05). There were significant differences in UA, FPG, UN, and HbA1C among the three groups (all *P* < 0.05). In addition, the duration of DM in the HUDR group and DR group was significantly different from that in the T2DM group (*P* < 0.05) (Table 1).

**Table 1:** Clinical baseline characteristics of the study subjects

Characteristics	T2DM (*N* = 100)	DR (*N* = 100)	HUDR (*N* = 100)	*P*_a_	*P*_b_	*P*_c_
Age (year)	56.46 ± 8.15	58.24 ± 7.82	58.38 ± 9.06	0.290	0.237	0.992
Gender (male/female)	46/54	58/42	51/49	0.119	0.572	0.394
BMI (kg/m^2^)	25.82 ± 3.54	26.74 ± 3.62	26.83 ± 3.55	0.164	0.114	0.983
DM duration (year)	5.16 ± 1.02	6.09 ± 2.23	6.21 ± 2.46	0.003	0.001	0.906
SBP (mm/Hg)	132.27 ± 15.50	128.92 ± 14.48	133.67 ± 17.06	0.289	0.804	0.084
DBP (mm/Hg)	78.40 ± 10.38	80.66 ± 11.24	79.35 ± 9.57	0.277	0.796	0.648
Smoking (yes/no)	38/62	43/57	35/65	0.565	0.769	0.310
Drinking (yes/no)	42/58	37/63	33/67	0.563	0.243	0.657
Hypertension (yes/no)	27/73	32/68	24/76	0.535	0.746	0.270
Coronary heart disease (yes/no)	31/69	28/72	25/75	0.757	0.431	0.749
UA (μmol/L)	276.68 ± 46.73	346.57 ± 42.58	434.98 ± 45.25	<0.001	<0.001	<0.001
FPG (mmol/L)	5.46 ± 0.74	5.79 ± 0.78	6.08 ± 0.92	0.013	<0.001	0.034
Fasting insulin (pmol/L)	35.21 ± 12.74	35.67 ± 13.82	37.08 ± 15.04	0.970	0.608	0.753
Fasting C-peptide (pmol/L)	720.53 ± 65.78	716.27 ± 84.31	734.32 ± 86.92	0.924	0.439	0.245
Creatinine (μmol/L)	72.69 ± 10.56	72.45 ± 11.21	73.21 ± 14.88	0.990	0.952	0.901
UN (mmol/L)	4.25 ± 1.06	4.72 ± 1.44	5.17 ± 1.48	0.036	<0.001	0.048
TC (mmol/L)	4.41 ± 1.87	4.34 ± 1.92	4.39 ± 2.02	0.965	0.997	0.982
TG (mmol/L)	1.62 ± 0.75	1.76 ± 0.81	1.81 ± 0.79	0.417	0.202	0.894
LDL-C (mmol/L)	2.64 ± 0.65	2.73 ± 0.57	2.70 ± 0.68	0.576	0.782	0.940
HDL-C (mmol/L)	1.20 ± 0.31	1.16 ± 0.28	1.22 ± 0.34	0.635	0.892	0.361
HbA1C (%)	8.36 ± 1.47	8.97 ± 1.68	9.56 ± 1.86	0.029	<0.001	0.036

Note: BMI: body mass index; DM: diabetes mellitus; SBP: systolic blood pressure; DBP: diastolic blood pressure; UA: uric acid; FPG: fasting plasma glucose; UN: urea nitrogen; TC: total cholesterol; TG: triglyceride; LDL-C: low density lipoprotein cholesterol; HDL-C: high density lipoprotein cholesterol; HbA1C: hemoglobin A1C. The measurement data were expressed as mean ± standard deviation or number of cases. Fisher’s exact test was employed for the comparative analysis of categorical variables. One-way ANOVA was applied for data comparisons among groups of continuous variables, followed by Tukey’s multiple comparisons test. *P* < 0.05 indicated statistical significance. *P*_a_: DR group compared with T2DM group; *P*_b_: HUDR group compared with T2DM group; *P*_c_: HUDR group compared with DR group.

### 3.2 Comparisons of serum VEGF, hs-CRP, and Cys-C levels

**Figure 1:**
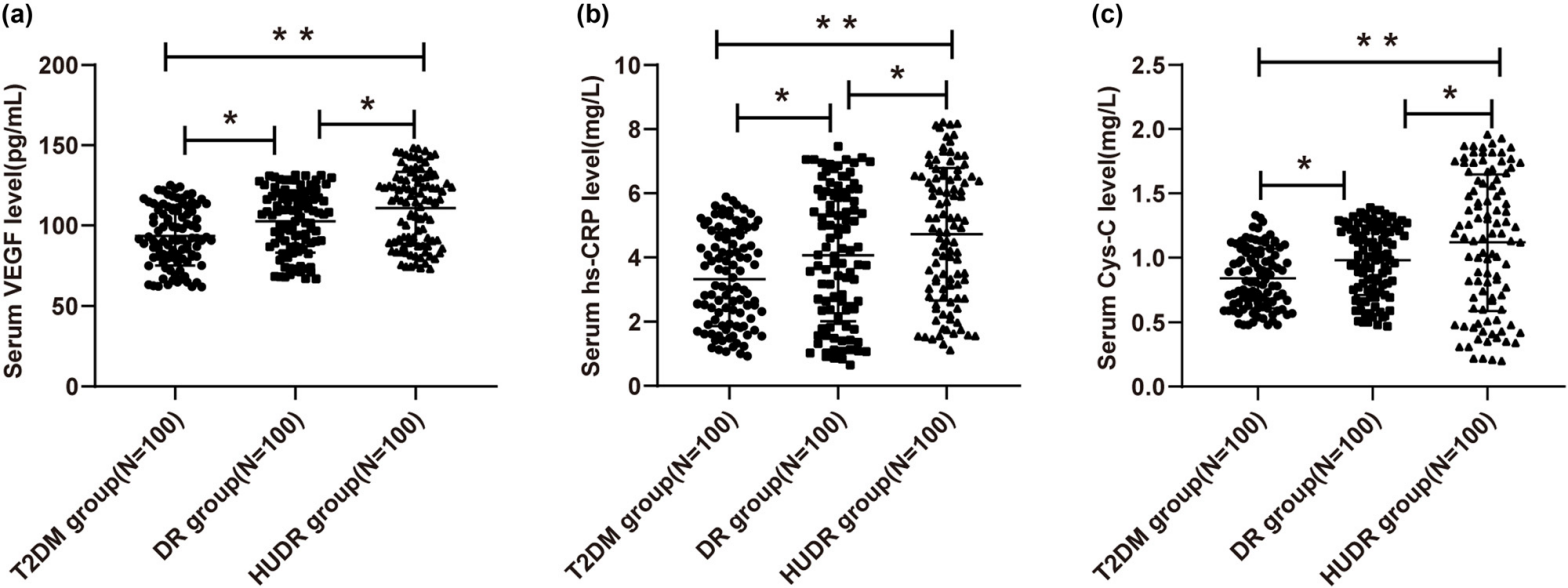
Comparisons of serum VEGF, hs-CRP, and Cys-C levels among groups. (a and b) Serum VEGF and hs-CRP levels in the T2DM group, DR group, and HUDR group were assessed by ELISA. (c) Serum Cys-C level was evaluated by immunoturbidimetry. Data were expressed as mean ± standard deviation. One-way ANOVA was applied for comparisons among groups, followed by Tukey’s multiple comparisons test. *P < 0.05, **P < 0.01.

Serum VEGF and hs-CRP levels of the T2DM group, DR group, and HUDR group were measured by ELISA. Serum VEGF levels in the T2DM group, DR group, and HUDR group were 93.37 ± 18.26 (pg/mL), 102.52 ± 19.44 (pg/mL), and 110.96 ± 22.53 (pg/mL), respectively. The differences in serum VEGF levels among the three groups were statistically significant, with the level in the HUDR group > DR group > T2DM group (*P* < 0.05) (Figure 1a). Serum hs-CRP levels in the T2DM group, DR group, and HUDR group were 3.32 ± 1.45 (mg/L), 4.06 ± 2.04 (mg/L), and 4.72 ± 2.07 (mg/L), respectively There were statistical differences among the three groups, with the level in the HUDR group > DR group > T2DM group (all *P* < 0.05) (Figure 1b). Serum Cys-C level was evaluated by immunoturbidimetry. Serum Cys-C levels in the T2DM group, DR group, and HUDR group were 0.84 ± 0.23 (mg/L), 0.98 ± 0.27 (mg/L), and 1.12 ± 0.53 (mg/L), respectively. There were statistical differences among the three groups, with the level in the HUDR group > DR group > T2DM group (all *P* < 0.05) (Figure 1c). Based on these, we speculated that VEGF, hs-CRP, and Cys-C might promote DR in DM patients and are closely related to DR progression.

### 3.3 Correlation analysis of serum UA level with VEGF, hs-CRP, and Cys-C levels in HUDR patients

**Figure 2:**
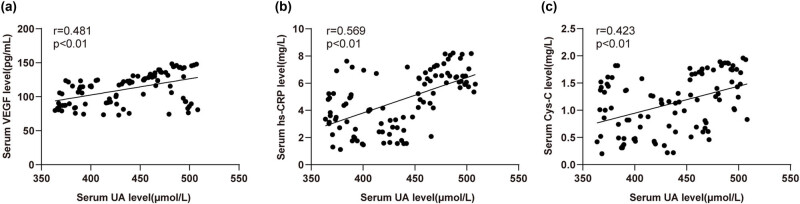
Correlation analysis between serum UA level with VEGF, hs-CRP, and Cys-C levels in HUDR patients. (a–c) Correlations between serum UA level with VEGF, hs-CRP, and Cys-C in HUDR patients. Panels (a)–(c) were analyzed by Pearson coefficient.

Next, the correlations between serum UA level and VEGF, hs-CRP, and Cys-C levels were analyzed by Pearson tests. Serum UA level in HUDR patients was positively correlated with serum VEGF, hs-CRP, and Cys-C levels (*r* = 0.481, 0.569, 0.423) (all *P* < 0.01) (Figure 2a–c). These results suggested that HUA might promote DR progression by up-regulating VEGF, hs-CRP, and Cys-C expression levels.

### 3.4 Diagnostic values of serum VEGF, hs-CRP, and Cys-C in HUDR patients

**Figure 3:**
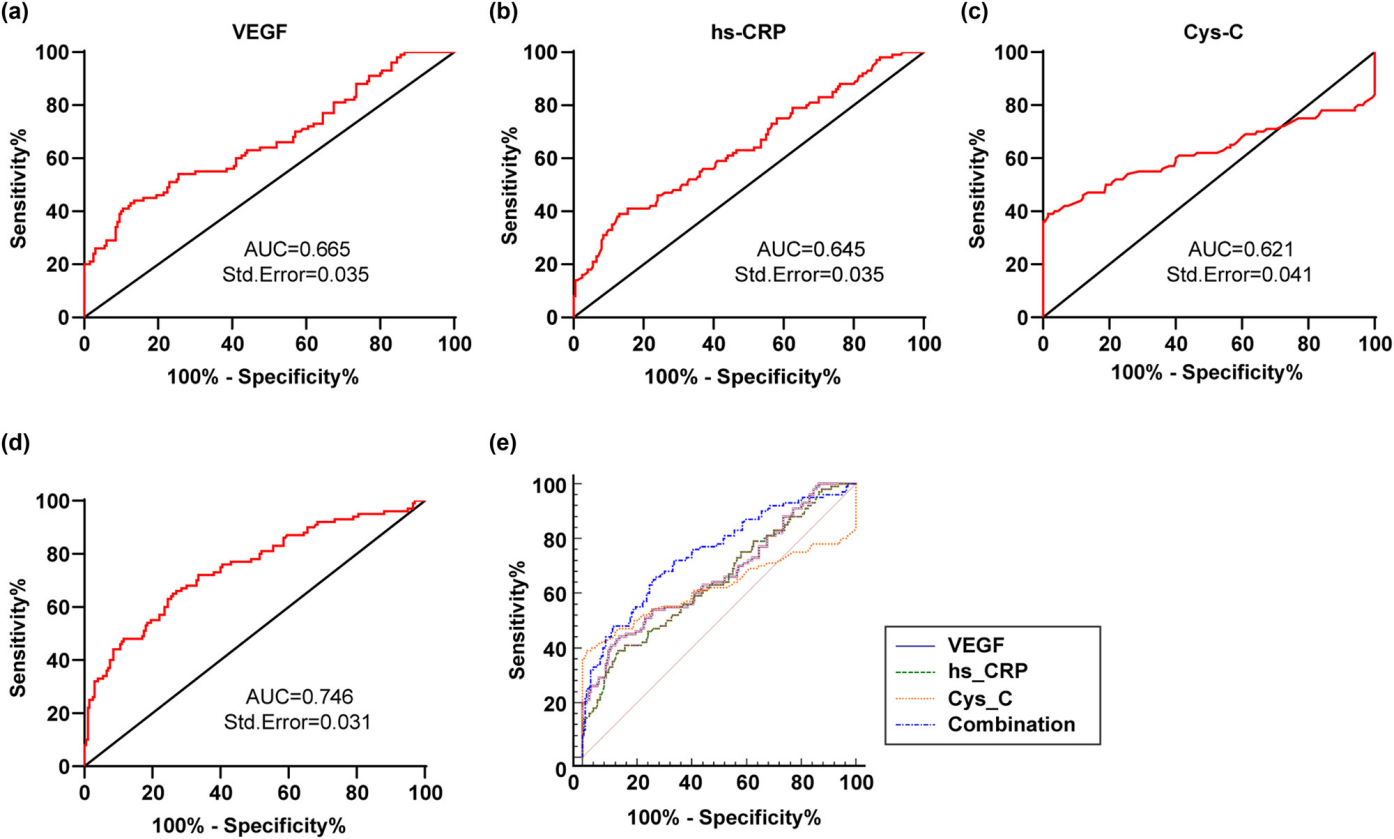
Diagnostic values of serum VEGF, hs-CRP, and Cys-C in HUDR patients. (a–d) Diagnostic efficacy of serum VEGF, hs-CRP, Cys-C and their combination in HUDR patients was evaluated using the ROC curve. (e) Differences in AUC were analyzed by MedCalc.

The diagnostic efficacy of serum VEGF, hs-CRP, and Cys-C for HUDR patients was evaluated using the ROC curve. The AUC of serum VEGF for HUDR diagnosis was 0.665, and the cut-off value was 121.30 (43.00% sensitivity and 87.50% specificity), indicating that serum VEGF level >121.30 pg/mL assisted in HUDR diagnosis (Figure 3a). The AUC of serum hs-CRP for HUDR diagnosis was 0.645, and the cut-off value was 5.895 (39.00% sensitivity and 87.00% specificity) (Figure 3b), indicating that serum hs-CRP level >5.90 mg/L assisted in HUDR diagnosis. The AUC of serum Cys-C for HUDR diagnosis was 0.621, and the cut-off value was 1.355 (39.00% sensitivity and 98.50% specificity) (Figure 3c). In addition, the AUC of VEGF, hs-CRP, and Cys-C combination for HUDR diagnosis was 0.746 (66.00% sensitivity and 73.00% specificity) (Figure 3d). Moreover, MedCalc analysis of AUC difference elicited that the diagnostic value of VEGF, hs-CRP, and Cys-C combination for HUDR diagnosis was higher than those of independent VEGF, hs-CRP, and Cys-C (Figure 3e).

### 3.5 Logistic multiple factor regression analysis

To explore the risk factors affecting HUDR, DM duration, UA, FPG, UN, HbA1C, VEGF, hs-CRP, and Cys-C were included in the binary logistic regression analysis model with HUDR occurrence as the dependent variable, and the statistically significant indicators in the univariate analysis as the independent variable. UA (*P* = 0.001, OR = 1.074, 95% CI: 1.052–1.096), FPG (*P* = 0.018, OR = 1.974, 95% CI: 1.122–3.473), HbA1C (*P* = 0.038, OR = 1.361, 95% CI: 1.017–1.822), VEGF (*P* = 0.048, OR = 0.975, 95% CI: 0.950–1.000), hs-CRP (*P* = 0.036, OR = 0.776, 95% CI: 0.613–0.984), and Cys-C (*P* = 0.017, OR = 0.184, 95% CI: 0.046–0.741) were independent risk factors for HUDR (Tables 2 and 3).

**Table 2:** Meaning and assignment method of variables

Factors	Meaning	Assignment
*y*	Whether HUDR occurred	*y* = 1 yes, *y* = 0 no
*x*_1_	DM duration	Continuous variable (year)
*x*_2_	UA	Continuous variable (μmol/L)
*x*_3_	FPG	Continuous variable (mmol/L)
*x*_4_	UN	Continuous variable (mmol/L)
*x*_5_	HbA1C	Continuous variable (%)
*x*_6_	VEGF	Continuous variable (pg/mL)
*x*_7_	hs-CRP	Continuous variable (mg/L)
*x*_8_	Cys-C	Continuous variable (mg/L)

**Table 3:** Logistic multivariate regression analysis of risk factors affecting DR patients

Variable	*P* value	OR	OR 95% CI
DM duration	0.965	1.005	0.811–1.245
UA	0.001	1.074	1.052–1.096
FPG	0.018	1.974	1.122–3.473
UN	0.959	0.991	0.702–1.399
HbA1C	0.038	1.361	1.017–1.822
VEGF	0.048	0.975	0.950–1.000
hs-CRP	0.036	0.776	0.613–0.984
Cys-C	0.017	0.184	0.046–0.741

### 3.6 Comparisons of PDR and NPDR in the HUDR group and DR group

DR patients were allocated into NPDR and PDR according to the results of ophthalmic examination and the severity of the disease. The incidence of PDR in the HUDR group and DR group was 42.00% (42/100) and 27.00% (27/100), respectively, and there was a difference between the two groups (*χ* ^2^ = 4.978, *P* < 0.05) (Table 4). The results indicated that HUA might promote DR progression.

**Table 4:** Comparisons of PDR and NPDR in the HUDR group and DR group

Group	*N*	NPDR	PDR
HUDR group	100	58	42
DR group	100	73	27
*χ* ^2^	4.978		
*P*	0.026		

## 4 Discussion

DR is the most prevalent complication of diabetes mellitus [1]. Increasing prevalence of DM warrants recognition of the factors related to asymmetric DR [36]. Increasing evidence has shown the close associations of HUA, VEGF, hs-CRP, and Cys-C with the development of T2DM and its complication DR [37–40]. This study elaborated that serum levels of VEGF, hs-CRP, and Cys-C were elevated in HUDR patients, and HUA might promote the progression of DR.

Raised serum UA levels are positively related to the development of metabolic diseases including T2DM, chronic kidney disease, and hypertension, and can be applied as an independent and strong risk factor for the renal function decline in T2DM individuals [41]. As reported in the literature, UA to high-density lipoprotein ratio (UHR) possesses an independent predictable function in DKI and can be utilized as a tool for diagnosing DKI [11]. In addition, Aktas et al. have demonstrated that UHR is significantly associated with HbA1C and FPG levels, which can be regarded as a promising predictive index for regulating T2DM male diabetes [9]. Our results revealed significant differences in UA, FPG, UN, and HbA1C among HUDR, DR, and T2DM patients, indicating their involvement in DR progression.

VEGF is key to the process of angiogenesis in retinal neovascularization during PDR and VEGF antagonists are widely administered for the therapy of ocular pathologies including PDR, age-related macular degeneration, retinal vessel occlusion, and diabetic macular edema [42]. Chronic systemic inflammation is identified as a related factor in metabolic syndrome and DM, and serum hs-CRP can be measured at very low levels, which may indicate stimulated inflammatory activity in the vessel wall [43]. Research over the last few years has suggested that high Cys-C concentration is directly related to inflammation and atherosclerosis, as well as the incidence of T2DM [44]. In the present study, we found significant differences in VEGF, hs-CRP, and Cys-C levels in T2DM, DR, and HUDR patients, with the levels highest in HUDR patients and lowest in T2DM patients. Changes in Cys-C levels assist in the early diagnosis and treatment of DR to some extent, and patients with high Cys-C levels and long diabetes courses are more likely to have DR [45]. The activation of cytokines including VEGF is responsible for DR development, and elevated CRP levels demonstrate vascular damage in diabetic patients, but the studies about the relationship between DR and CRP level are controversial [35]. To conclude, VEGF, hs-CRP, and Cys-C might promote DR progression in DM patients. Moreover, we conducted Pearson analysis and discovered that the level of UA had positive correlations with the levels of VEGF, hs-CRP, and Cys-C in HUDR patients. Consistently, at baseline, with the increase of UA levels, DM subjects were more likely to have significantly higher CRP and elevated UA levels were associated with increased risks of DM incidence [46]. In patients with diabetic macular edema, intravitreal UA is correlated with intravitreal VEGF, and the role of UA in DR pathogenesis and progression should be considered [47]. In brief, HUA might promote the progression of DR by facilitating the levels of VEGF, hs-CRP, and Cys-C.

Next, we plotted ROC curves to evaluate the diagnostic efficacy of VEGF, hs-CRP, and Cys-C in HUDR and discovered that VEGF level >121.30 pg/mL, hs-CRP level >5.90 mg/L, and Cys-C level > 1.355 mg/L assisted in the diagnosis of HUDR, with their combination manifesting higher diagnostic values than the levels alone. Previous studies also reported the diagnostic values of VEGF, hs-CRP, and Cys-C in DR or DM. Circulating VEGF has a high diagnostic value in DR, with the potential to be its early diagnostic marker [48]. In T2DM patients with proteinuria, the ratio of TG/Cys-C has certain predictive values for diabetic kidney disease diagnosis [32]. However, less attention was paid to their combination diagnostic values in HUDR, and we were the first to state the higher combination diagnostic values. Furthermore, we conducted binary logistic regression analysis and elaborated that UA, HbA1C, VEGF, hs-CRP, and Cys-C were independent risk factors for HUDR. The management of metabolic risk factors, especially UA, HbA1c, TG, and SBP is advised to be performed in the early stage of DM [49]. UA has been proposed as an independent risk factor of DR [17]. Cys-C concentration was an independent predictor for long-term mortality and major adverse cardiac events in elder patients with acute coronary syndrome combined with diabetes mellitus [44]. Furthermore, we assigned DR patients to NPDR and PDR groups and found that the incidence of PDR in HUDR and DR patients was 42.00% (42/100) and 27.00% (27/100), respectively. Asymptomatic HUA should be considered a risk factor for the induction and progression of DR [32]. Briefly, HUA might promote DR progression.

In summary, this study analyzed the differences of serum VEGF, hs-CRP, and Cys-C in type 2 DR patients with HUA and normal UA levels, and their relationships between UA, which was helpful to pay more attention to UA levels in T2DM patients in clinical work, to reduce the occurrence and development of DR. Moreover, in clinical diagnosis and treatment of T2DM patients, the serum VEGF, hs-CRP, and Cys-C levels should be paid close attention. For patients with high risk, regular screening should be carried out, and preventive intervention measures should be used to play a positive role in diagnosis and condition monitoring, thus improving patient prognosis. It is also of clinical significance for guiding clinical doctors in DR diagnosis and treatment. However, the pathophysiological mechanisms of the correlations between UA with VEGF, hs-CRP, and Cys-C were not fully discussed. In addition, the mechanism that HUA might affect the occurrence and development of DR was not further confirmed. These are the direction for our future studies.
